# Detoxification of aflatoxin M_1_ by eight lactic acid bacteria strains, alone or in combination, as viable and heat-killed cells in PBS, MRS broth, and Karish cheese

**DOI:** 10.1038/s41598-026-62660-7

**Published:** 2026-08-10

**Authors:** Rafaat M. Elsanhoty, Mohammed Aladhadh, Mahmoud A. Al-Saman, Asmaa Abdella, Khaled M. Aboshanab, Samar S. Mabrouk

**Affiliations:** 1 Department of Industrial Biotechnology, Faculty of Biotechnology, University of Sadat City, Sadat City, 22857/79 Egypt; 2 Department of Food Science and Human Nutrition, College of Agriculture and Food, Qassim University, 51452 Buraydah, Saudi Arabia; 3 Department of Microbiology and Immunology, Faculty of Pharmacy, Ain Shams University, Cairo, 11566 Egypt; 4 Department of Microbiology, Faculty of Pharmacy, Ahram Canadian University (ACU), 6th October City, Giza, 12566 Egypt

**Keywords:** Aflatoxin M_1_, Lactic acid bacteria, Detoxification, Karish cheese, Food safety, Biotechnology, Microbiology

## Abstract

Aflatoxin M1 (AFM1) in milk is a major public health concern. It is a hydroxylated metabolite of aflatoxin B1 (AFB1), formed in the liver of animals after ingestion of contaminated feed and subsequently excreted into milk. Biological techniques could be used to combat this hepatotoxic and carcinogenic toxin. Therefore, this study evaluated the ability of eight lactic acid bacteria (LAB) strains to reduce AFM1 levels in phosphate-buffered saline (PBS), MRS broth, and Karish cheese using viable cells, while heat-inactivated cells, rendered non-viable by thermal treatment, were evaluated only in PBS. Eight standard LAB strains were tested, including *Lactobacillus acidophilus (L. acidophilus)* ATCC 4356*, Lactobacillus johnsonii (L. johnsonii)* ATCC 3320*, Lactobacillus salivarius (L. salivarius)* TISTR 390*, Lactobacillus bulgaricus (L. bulgaricus)* EMCC-3*, Lactobacillus paracasei (L. paracasei)* TISTR 453*, Lactobacillus helveticus (L. helveticus*) LH-BO2*, Streptococcus thermophilus (S. thermophilus)* EMCC-2, and *Bifidobacterium animalis subsp. lactis (B. animalis subsp. lactis)* Bb12. Viable cells exhibited strong detoxification efficiency, with *L. acidophilus* ATCC 4356 achieving the highest AFM1 reduction (≈76%), followed by *B. animalis subsp. lactis* Bb12 and *L. salivarius* TISTR 390. Heat-killed counterparts showed minimal activity, confirming the essential role of metabolic processes in toxin removal. In MRS broth, a significant relationship was observed between pH decline and AFM1 degradation, where up to 80% reduction occurred at pH 3.0 after 20 days, emphasizing the influence of acidic and enzymatic conditions. Application in Karish cheese demonstrated the practical relevance of LAB under real food conditions. Mixed cultures of *S. thermophilus* EMCC-2, *L. bulgaricus* EMCC-3, and *L. acidophilus* ATCC 4356 or *L. salivarius* TISTR 390 reduced AFM1 by 74–78% after 20 days at 4 °C, accompanied by gradual acidification during ripening. In conclusion, these findings demonstrate that lactic acid bacteria (LAB), particularly multi-strain systems, effectively reduce aflatoxin M₁ (AFM₁) levels in dairy matrices.

**Supplementary Information:**

The online version contains supplementary material available at 10.1038/s41598-026-62660-7.

## Introduction

AFM1 is one of the most hazardous mycotoxins found in milk and dairy products, originating as a hydroxylated metabolite of aflatoxin B₁ produced by *Aspergillus flavus* and *A. parasiticus*. Due to its heat stability and resistance to conventional pasteurization or fermentation processes, AFM1 poses a persistent risk to consumer health^1,2^. Chronic exposure to AFM1, even at low concentrations, has been associated with hepatotoxicity, immunosuppression, and carcinogenic effects^3^, leading to stringent regulatory limits worldwide. According to the International Agency for Research on Cancer^4^, AFM1 is classified as a group 1 human carcinogen.

The presence of this toxin in dairy products not only threatens food safety but also affects international trade and public confidence in dairy quality. AFM1 has been confirmed to have cytotoxic and carcinogenic effects in many species, although it is about 10 times less dangerous than AFB1^5^. In recent years, biological detoxification approaches have gained increasing attention as promising alternatives to chemical and physical methods, which often compromise food’s dietary and sensory properties. Among the biological agents investigated for this purpose, *lactic acid bacteria (LAB)* have shown significant potential for binding, degrading, or sequestering AFM1 through various mechanisms, including cell wall adsorption, enzymatic modification, and metabolic transformation^1^. LAB is commonly recognized as safe (GRAS) and has long been used as a starter culture in fermented dairy products, making them ideal candidates for natural detoxification strategies^6^.

Probiotic-based microbial food supplements have been shown to benefit both the body and the mind, including increased immunity, decreased lactose intolerance, a lower risk of cancer, improved intestinal tract health, pathogen antagonism, and increased nutrient synthesis and bioavailability^7^. A new technique for mycotoxin-free food is cold plasma, which produces reactive oxygen and nitrogen species. Physical methods such as washing, dehulling, aqueous extraction, cleaning, and milling have been shown to partially reduce mycotoxin levels in grains^8,9^. The amounts of AFs and fumonisin in tortillas were considerably decreased by alkaline cooking^10^. Fermentation has been used for centuries to preserve food, and research has demonstrated that LAB prevents the growth of mold and the creation of amyloid^11^

AFM1 was selected as the target toxin because it is the major hydroxylated metabolite of AFB₁ detected in milk and dairy products, representing a significant food safety concern for consumers. The present study evaluates the ability of eight lactic acid bacteria (LAB) strains, individually and in combination, to reduce AFM1 levels under different experimental conditions, including phosphate-buffered saline, MRS broth, and a real dairy matrix (Karish cheese). Previous studies have reported the potential of LAB to reduce aflatoxins in food systems, including comparative and multi-strain approaches^12,13^. However, limited research has investigated direct head-to-head comparisons of a larger number of LAB strains under standardized conditions, particularly in Karish cheese. Therefore, this study aimed to provide a systematic comparative assessment of eight LAB strains and to evaluate the effects of cell viability and pH changes during incubation and storage.

## Materials and methods

### Chemicals

The AFM1 standard was supplied by Sigma (St. Louis, MO, USA). HPLC-pure solvents were provided by Merk (Darmstadt, Germany). The AFM1 immune-affinity columns were provided by VICAM (Watertown, MA, USA). The water was cleaned using a Milli-Q water purification system (Baunstead, Dubuque, USA).

### Bacterial strains and culture conditions

Eight standard probiotic strains obtained from International Culture Collections (Table 1) were selected for their industrial relevance and reported effects on food mutagens. Cultivation was performed on De Man, Rogosa, and Sharpe (MRS; Difco Laboratories, USA) agar under optimal temperature and aeration conditions. Anaerobic strains were maintained in Oxoid anaerobic jars, while fully developed colonies were stored at 4 °C and sub-cultured monthly. For long-term preservation, bacterial cultures were cryostored at -80°C in sterile 90% glycerol stock solution used as a cryoprotective agent. *Lactobacilli* strains were cultured in MRS broth, and *Bifidobacteria* strains in MRS supplemented with cysteine. After 24 h of incubation at 37 °C, cultures were centrifuged at 3500 × g for 10 min, and cells were washed with sterile phosphate-buffered saline (PBS, pH 7.3) for AFM1 detoxification assays^14^.

**Table 1:** List, sources, and oxygen requirement of the eight standard LAB strains used in this study.

Strain	Source	Oxygen requirement
*Lactobacillus helveticus* LH-BO2	1	Facultatively anaerobic/aerotolerant anaerobe
*B. animalis subsp. lactis* Bb12	1	Anaerobic (oxygen-tolerant)
*Lactobacillus salivarius* TISTR 390	2	Facultatively anaerobic/aerotolerant anaerobe
*Lactobacillus paracasei *TISTR 453	3	Facultatively anaerobic/aerotolerant anaerobe
*Lactobacillus johnsonii *ATCC 3320	4	Microaerophilic, aerotolerant anaerobe
*Lactobacillus acidophilus* ATCC 4356	4	Microaerophilic, aerotolerant anaerobe
*Streptococcus thermophilus* EMCC-2	2	Facultatively anaerobic
*Lactobacillus bulgaricus* EMCC-3	2	Facultatively anaerobic to microaerophilic

**1**, Chr. Hansen-Denmark;**2**, Egyptian Microbial Culture Collection (EMCC) at Cairo Microbiological Resources Centre (Cairo, MIRCEN), Faculty of Agriculture, Ain Shams University, Cairo, Egypt; **3**, Thailand Institute of Scientific and Technological Research, Bangkok, Thailand; **4**, The American Type Culture Collection (ATCC).

### Milk powder

A total of 100 mL of demineralized water was added to a flask holding 10 g of milk powder. After 10 min of stirring, the mixture was centrifuged for an additional 10 min at 20°C and 3500 × g. About 100 mL of skim milk was utilized in the following HPLC analysis to detect AFM1 after the top fatty layer was eliminated by centrifugation as previously described by the Association of Official Analytical Collaboration International (AOAC)^15^.

### Preparation of Karish cheese

Skim milk was used for Karish cheese manufacture because it represents the conventional substrate for this product and provides a simplified dairy matrix with reduced fat-related interference during AFM₁ analysis. The starter culture combinations were designed to compare a conventional dairy starter culture with formulations supplemented with LAB strains that demonstrated superior AFM₁ removal performance in the preliminary screening assays. Karish cheese was prepared following Ahmed et al. by reconstituting 1 kg skim milk powder in 9 L of water^16^. Milk was heated to 90 °C for 15 min, cooled to 47 °C, and inoculated with three different starter cultures: Starter #1 (*S. thermophilus* + *L. bulgaricus*, 1:1), Starter #2 (*S. thermophilus* + *L. bulgaricus* + *L. acidophilus* ATCC 4356, 1:1:1), and Starter #3 (*S. thermophilus* + *L. bulgaricus* + *L. salivarius* TISTR 390, 1:1:1). After coagulation (pH 4.5–4.7), curd was drained, salted (0.5%), cut into cubes, wrapped, and stored at 4 °C for 20 days. Samples were collected at days 5, 10, 15, and 20 for AFM1 analysis, with some stored at − 20 °C for further tests.

After coagulation (pH 4.5–4.7), the curd was drained, salted (0.5%), cut into cubes, wrapped, and stored at 4°C for 20 days. Samples were collected at days 5, 10, 15, and 20 for AFM₁ analysis. Portions of the collected samples were subsequently preserved at − 20°C until analysis to prevent further physicochemical and microbiological changes. AFM₁ standard solution was added to the reconstituted skim milk to obtain a final concentration of 50 µg/L before heat treatment and cheese manufacture. The contaminated milk was thoroughly mixed to ensure homogeneous toxin distribution. Subsequently, the milk was heated, cooled, inoculated with the respective starter cultures, and processed into Karish cheese. AFM₁ concentrations were monitored during refrigerated storage and expressed relative to the initial spiked concentration.

### Inoculum preparation

The microorganisms listed in Table 1 were grown in 100 mL of MRS broth (Oxoid CM 359, London, UK) at 37°C daily. *S. thermophilus* was grown in 25 mL of M17 broth (Oxoid CM817, London, UK) at 37°C. The Neubauer hemocytometer counting was used to assess the bacterial growth status for 2 h until a consistent growth pattern appeared. Then the concentration of each bacterial inoculum was maintained at 1 × 10^^6^ cells/mL after adjustment.

### Assessment of AFM₁ removal by viable and heat-treated bacterial cells in PBS

To evaluate the ability of viable and heat-treated bacterial cells to remove AFM₁, each bacterial culture was grown under the appropriate conditions as described above. For the binding assay, 2 mL of each pure culture (approximately 10⁶ CFU mL⁻^1^) was suspended in 98 mL of sterile phosphate-buffered saline (PBS, pH 7.3) artificially contaminated with AFM₁ at a final concentration of 50 µg L⁻^1^. For the preparation of heat-treated cells, bacterial suspensions were boiled in 4 mL PBS for 1 h to ensure complete loss of viability according to the method of Haskard et al.^17^. PBS was used as a non-nutritive medium to minimize bacterial growth and evaluate AFM₁ removal under controlled conditions independent of metabolic activity.

Viable cells were maintained under the same conditions without heat treatment. The contaminated suspensions were incubated at 37 °C for 24 h. During the 24 h incubation period, samples (2 mL) were collected from each bacterial suspension at 2 h intervals. The collected samples were centrifuged at 3500 × g for 10 min, and the supernatants were subsequently analyzed for residual AFM₁ concentration. Cell-free PBS artificially contaminated with AFM_1_ (50 µg L^−1^) was included as a positive control to assess toxin stability in the absence of bacterial cells. In addition, bacterial suspensions maintained in AFM₁-free PBS were used as negative controls throughout the experiment to monitor bacterial viability. Following incubation, all samples were centrifuged at 3500 × g for 10 min. The supernatants were collected and analyzed for residual AFM₁ concentration. AFM₁ removal efficiency was calculated based on the difference between the initial and residual toxin concentrations.

### Assessment of AFM_1_ removal by viable bacterial strains in contaminated MRS broth

The ability of viable bacterial strains to reduce AFM₁ in MRS broth during refrigerated storage was evaluated using MRS broth artificially contaminated with AFM_1_ at a concentration of 50 µg L^−1^. Approximately 0.5 mL of each bacterial inoculum (≈10⁶ CFU mL^−1^) was added to the contaminated medium and mixed thoroughly. The inoculated cultures were stored at 4 °C, and samples were collected after 1, 5, 10, 15, and 20 days of storage. At each sampling interval, bacterial cultures were centrifuged at 3500 × g for 10 min, and the resulting supernatants were used for AFM_1_ etermination and pH measurement.

### Extraction and preparation of samples for AFM₁ analysis

The concentration of AFM₁ was determined according to the AOAC (2005) method. Briefly, 2 g of sample were extracted with 40 mL dichloromethane for 15 min. The extract was filtered, and 10 mL of the filtrate were evaporated at 60 °C to dryness. The residue was reconstituted with 0.5 mL methanol, 0.5 mL PBS buffer, and 1 mL hexane. The mixture was centrifuged at 3500 × g for 15 min at 15 °C. After removal of the upper hexane layer, 100 µL of the lower phase were diluted with 400 µL deionized water (Fluka, Dresden, Germany). The resulting solution was used for AFM_1_ quantification by HPLC analysis.

### Determination of AFM1 by high-performance liquid chromatography (HPLC)

AFM₁ analysis was performed using the VICAM immunoaffinity column method according to Mwanza et al.. Samples (20 g) were extracted with 80 mL dichloromethane and centrifuged at 3500 × g for 10 min to remove fat^18^. A 50 mL aliquot of the resulting extract was then purified through the immunoaffinity column, and AFM_1_ was eluted with methanol and a methanol–acetonitrile mixture (3:2, *v/v*) before quantification. Eluates were diluted 1:3 with deionized water, filtered, and 10 µL injected into an HPLC system (Agilent 1100) equipped with a fluorescence detector (excitation 360 nm, emission 430 nm). Separation was performed on a C_18_ column using an isocratic mobile phase of acetonitrile, methanol, and water (16:22:62, v/v/v). A calibration curve was generated from AFM1 standards in acetonitrile.

#### Statistical and cluster analysis

All experimental data were expressed as the mean ± standard deviation (SD) of triplicate determinations. Statistical significance among treatments was analyzed using one-way analysis of variance (ANOVA) followed by Duncan’s multiple range test at a significance level of p < 0.05. To explore the relationships among the tested LAB strains based on their AFM₁ removal capacity in MRS broth, hierarchical cluster analysis (HCA) was performed using Ward’s linkage method and Euclidean distance as a measure of similarity. The resulting dendrogram was used to visualize clustering patterns and identify groups of strains exhibiting similar AFM₁ detoxification performance. All statistical analyses were performed using IBM SPSS Statistics software (Version 16, IBM Corp., Armonk, NY, USA).

## Results

### Characterization of lactic acid bacteria strains utilized for aflatoxin M_1_ detoxification

As displayed in Table 1, eight LAB strains were employed in the current study, highlighting their sources and oxygen requirements. The selected strains represent a diverse taxonomic and physiological spectrum of LAB, encompassing both *Lactobacillus* and *Streptococcus* genera, in addition to *Bifidobacterium*. This diversity was intentionally included to provide a comprehensive evaluation of their detoxification potential against AFM1 under varying environmental and metabolic conditions. The strains originated from internationally recognized microbial repositories, including Chr. Hansen (Denmark), the Egyptian Microbial Culture Collection (EMCC, Ain Shams University, Cairo, Egypt), the Thailand Institute of Scientific and Technological Research (TISTR), and the American Type Culture Collection (ATCC). Such a broad range of sources ensured the inclusion of strains adapted to different ecological niches and fermentation practices, thereby increasing the representativeness of the study.

### Detection of aflatoxin M_1_ in dairy matrix by high-performance liquid chromatography (HPLC)

Fig. S1 (supplementary data) illustrates the HPLC chromatograms used for the detection and quantification of AFM1. Chromatogram (A) represents the AFM1 standard solution at a concentration of 3.6 ng mL^−1^, showing a distinct and sharp peak at a retention time of approximately 12.87 min, which confirms the elution position of pure AFM1 under the applied chromatographic conditions. In contrast, chromatogram (B) corresponds to a Karish cheese sample, in which the same characteristic AFM1 peak appeared at the same retention time (12.87 min), indicating the presence of the toxin in the analyzed dairy matrix. However, additional smaller peaks observed at 2.154 min and 5.798 min correspond to other naturally occurring compounds or matrix interferences commonly found in fermented dairy products.

The similarity in retention time between the standard and the sample confirms the identity of AFM_1_ in the tested cheese. The relatively lower peak area in the sample chromatogram compared with the standard demonstrates a reduced AFM_1_ concentration, likely due to the binding or degradation effect of LAB used during fermentation. This finding highlights the effectiveness of LAB strains in mitigating AFM1 contamination through adsorption or biotransformation mechanisms during cheese processing and storage.

### Comparison of AFM_1_ detoxification efficiency among viable LAB strains

The data presented in Table 2 evaluate the efficiency of various viable LAB strains in reducing AFM_1_ concentration from PBS. The initial concentration of AFM_1_ was 50 µg/L, and changes in its residual level were recorded after 4, 8, 16, and 24 h of incubation at 37 °C. Across all LAB strains tested, a progressive reduction in AFM1 concentration was observed with increasing incubation time. This indicates that viable LAB cells possess the ability to interact with and remove AFM1, most likely through adsorption to cell wall components or by partial metabolic transformation.

**Table 2:** Effect of viable lactic acid bacteria strains on aflatoxin M1 (AFM1) reduction in phosphate buffer saline (PBS).

Viable strain^a^	Concentration of AFM1(µg/L)				
	4 hours^b^	8 h	16 h	24 h	% AFM1 removal after 24 h
*L. helveticus* LH-BO2	35.2 ± 2.2	25.2 ± 2.3	19.9 ± 3.1	15.8 ± 1.5	68.4 ± 1.43^bc^
*B. animalis subsp. lactis* Bb12	35.5 ± 2.7	37.9 ± 2.7	38.9 ± 2.9	14.96 ± 0.5	70.1 ± 0.89^b^
*L. salivarius* TISTR 390	36.8 ± 2.1	24.3 ± 2.3	19.8 ± 4.1	15.4 ± 1.3	69.2 ± 2.23^b^
*L. paracasei* TISTR 453	37.4 ± 3.1	34.8 ± 3.7	31.1 ± 1.3	16.1 ± 0.1	67.8 ± 1.54^c^
*L. johnsonii* ATCC 3320	38.8 ± 2.2	28.8 ± 2.3	18.7 ± 2.1	16.2 ± 3.1	67.6 ± 1.67^c^
*L. acidophilus* ATCC 4356	31.5 ± 3.1	21.1 ± 3.6	18.5 ± 2.3	11.8 ± 0.46	76.4 ± 2.04^a^
*S. thermophilus* EMCC-2	36.6 ± 0.55	28.5 ± 1.89	22.9 ± 0.78	17.6 ± 0.57	64.8 ± 0.61^d^
*L. bulgaricus *EMCC-3	35.1 ± 0.69	29.1 ± 1.32	21.8 ± 0.35	15.4 ± 0.89	69.2 ± 0.85^b^

The initial AFM1concentration was 50 µg/L.

Mean ± standard deviation (SD) of triplicate samples.

Different superscript letters in the same column indicate a significant difference at p < 0.05.

^a^Viable bacteria were incubated in PBS (4 mL) at 37°C for 1 h.

^b^Incubation period.

After 24 h, all strains achieved substantial AFM1 reduction, ranging from 64.8 to 76.4%. Among the tested LAB, *L. acidophilus* ATCC 4356 exhibited the highest detoxification efficiency, reducing the AFM1 concentration to 11.8 µg/L, equivalent to76.4% removal. This strain-dependent detoxification pattern is consistent with previous findings by Panwar et al., who reported significant AFM₁ reduction by probiotic *Lactobacillus* strains in an in vitro digestion model^19^. Both *B. animalis subsp. Lactis* Bb12 and *L. salivarius* TISTR 390 also demonstrated strong performance with 70.1 and 69.2% removal, respectively. In contrast, *S. thermophilus* showed the lowest reduction (only 64.8%) after 24 h, suggesting comparatively weaker binding affinity or fewer active cell wall sites for AFM1 interaction. At the early incubation stage (4 h), the AFM1 concentration remained relatively high for all strains, with values between 31.5 and 38.8 µg/L, corresponding to 22–37% removal. After 8 h, a marked destabilization was observed, especially for *B. animalis subsp. lactis* (37.9 ± 2.7 µg/L removed). However, between 16 and 24 h, the reduction rate tended to stabilize, suggesting that the binding capacity of bacterial cells reached equilibrium-possibly due to saturation of adsorption sites or reversible toxin release at prolonged exposure.

The transient increase in residual AFM₁ concentration observed for B. lactis Bb12 during the first 16 h may indicate a reversible adsorption process. AFM₁ binding to bacterial cell-wall components is known to involve weak non-covalent interactions, which may permit temporary desorption before a more stable adsorption equilibrium is achieved at longer incubation times.

### Residual AFM1 concentrations after incubation with Heat-killed LAB

Table 3 shows residual AFM1 concentrations (µg/L) after incubation of heat-killed LAB strains with an initial AFM1 level of 50 µg/L, measured at 4, 8, 16, and 24 h. Bacteria were boiled in PBS for 1 h (heat-treated). Triplicate means ± SD are presented. Heat-killed cells produced only modest AFM1 reductions over 24 h. Similar observations were reported by Panwar et al., who showed that non-viable or heat-treated cells exhibit markedly reduced AFM₁ binding compared to viable probiotic cultures, highlighting the importance of intact cell surface structure^17,19^. Residual AFM1 at 24 h ranged from 41.3 to 46.7 µg/L, corresponding to 6.6–17.4% removal of the initial 50 µg/L. These removal levels are substantially lower than those achieved with viable LAB in the previous table (where removals were ~ 65–76% after 24 h). This indicates that viability (or the cell state before heat treatment) strongly affects AFM1 removal under the tested conditions.*L. acidophilus*, *L. helveticus,* and *L. bulgaricus* performed best among heat-killed strains (~ 16–17% removal), while *B. animalis subsp. lactis*, *L. paracasei,* and *L. johnsonii* showed minimal effect (~ 6–8%).

**Table 3:** Effect of heat-killedLABstrains on AFM1 removal from phosphate buffer saline (PBS).

Heat-killedstrain^a^	concentration of AFM1(µg/L)				
	4 hours^b^	8 h	16 h	24 h	% AFM1 removal after 24 h
*L. helveticus* LH-BO2	44.8 ± 0.55	43.9 ± 0.67	42.9 ± 0.28	41.9 ± 0.39	16.2 ± 1.46^a^
*B. animalis subsp. lactis *Bb12	47.9 ± 2.8	46.7 ± 0.11	46.5 ± 0.27	46.7 ± 0.16	6.6 ± 1.89^c^
*L. salivarius* TISTR 390	45.9 ± 2.3	44.4 ± 0.34	44.2 ± 0.24	43.0 ± 0.21	14.0 ± 3.21^b^
*L. paracasei* TISTR 453	47.9 ± 1.7	46.9 ± 0.45	46.7 ± 0.34	46.2 ± 0.56	7.6 ± 0.89^c^
*L. johnsonii* ATCC 3320	47.9 ± 2.8	46.9 ± 4.6	46.7 ± 0.14	46.4 ± 0.23	7.2 ± 0.91^c^
*L. acidophilus* ATCC 4356	42.4 ± 1.7	41.0 ± 0.67	41.2 ± 0.11	41.3 ± 0.46	17.4 ± 1.46^a^
*S. thermophilus *EMCC-2	44.1 ± 0.33	43.8 ± 0.32	43.8 ± 0.15	43.2 ± 0.15	13.6 ± 2.62^b^
*L. bulgaricus *EMCC-3	44.6 ± 0.55	42.9 ± 067	42.3 ± 0.28	41.9 ± 0.29	16.2 ± 1.09^a^

The initial AFM1concentration was 50 µg/L.

Mean ± standard deviation of triplicate samples.

Different superscript letters in the same column indicate a significant difference at p < 0.05.

^a^Bacteria were boiled in PBS (4 mL) for 1 h (heat-treated).

^b^Incubation period.

The change from 4 to 24 h is small for all strains (typical reductions of ~ 2–6 µg/L over 20 h). For example, *L. helveticus* decreased from 44.8 µg/L (4 h) to 41.9 µg/L (24 h) (a net removal of 2.9 µg/L, ~ 5.8% absolute). The near-plateau behavior indicates rapid attainment of equilibrium between AFM1 in solution and binding sites on heated cells, with little progressive removal after early contact. Heat-killed LAB are poor standalone solutions for AFM1 removal under the tested conditions (max ~ 17% removal). For meaningful decontamination, viable cultures or other interventions are required.

### Effect of incubation period and pH variation on the reduction of AFM1 by LAB strains in MRS broth

The data displayed in Table 4 summarizes the relationship between pH variation and AFM1 degradation by different lactic acid bacteria (LAB) strains during incubation in MRS broth for 1, 5, 10, 15, and 20 days. The initial AFM1 concentration was 50 µg/L. Both AFM1 reduction and acidification of the medium (pH decline) were monitored as indicators of bacterial metabolic activity and detoxification potential. All LAB strains exhibited a progressive decrease in AFM11 concentration with increasing incubation time, accompanied by a steady decline in pH values from approximately 4.5 on day 1 to around 3.0 by day 20. This simultaneous decline suggests that acid production and active metabolism play crucial roles in enhancing AFM1 removal. After 20 days, the most efficient strain was *L. acidophilus* ATCC 4356, which reduced AFM1 concentration from 50 µg/L to 10 µg/L, corresponding to an 80% removal rate. *L. salivarius* followed closely with 79.8% removal, while *B. animalis subsp. lactis* and *L. paracasei* achieved approximately 75–76% reduction. The least effective strain was *S. thermophilus* with 70.4% removal, though still significant compared to the initial concentration.

**Table 4:** Effect of LAB strains and pH changes during incubation periods on the reduction of AFM1 in MRS broth.

Strain	Incubation time										% AFM1 removal
	1 D		5 D		10 D		15 D		20 D		
	pH#	AFM1^@^	pH	AFM1	pH	AFM1	pH	AFM1	pH	AFM1	
*L. helveticus* LH-BO2	4.5	40.1 ± 1.2	4.3	34.1 ± 1.8	3.9	25.1 ± 1.5	3.6	18.4 ± 1.2	3.0	13.2 ± 4.5	73.6 ± 2.04^c^
*B. animalis subsp. lactis *Bb12	4.7	39.8 ± 2.3	4.2	32.0 ± 2.8	3.9	24.5 ± 2.8	3.5	18.0 ± 2.3	3.0	11.9 ± 1.9	76.2 ± 1.90^b^
*L. salivarius* TISTR 390	4.4	39.9 ± 2.7	4.0	25.9 ± 1.1	3.9	17.6 ± 4.1	3.4	14.2 ± 3.4	3.0	10.2 ± 5.2	79.8 ± 1.04^a^
*L. paracasei* TISTR 453	4.3	38.6 ± 3.1	4.0	34.9 ± 2.3	3.8	28.7 ± 1.9	3.4	20.0 ± 3.3	3.0	12.1 ± 1.8	75.8 ± 0.94^b^
*L. johnsonii* ATCC 3320	4.6	38.9 ± 3.2	4.2	30.0 ± 1.8	3.8	25.2 ± 1.5	3.4	18.6 ± 4.1	3.1	13.2 ± 2.5	73.6 ± 1.09^c^
*L. acidophilus* ATCC4356	4.5	31.6 ± 3.5	4.0	23.9 ± 2.66	3.8	16.0 ± 1.89	3.5	13.1 ± 2.2	3.0	10.0 ± 2.5	80.0 ± 2.10^a^
*S. thermophilus* EMCC-2	4.3	36.6 ± 0.97	4.0	28.3 ± 1.3	3.7	22.1 ± 3.3	3.5	19.0 ± 4.2	3.0	14.9 ± 2.4	70.4 ± 1.50^de^
*L. bulgaricus* EMCC-3	4.5	36.0 ± 1.4	4.0	26.1 ± 3.4	3.6	21.9 ± 2.7	3.4	19.0 ± 1.2	3.0	13.9 ± 2.8	72.2 ± 0.71^cd^

^**#**^ measured pH value.

^**@**^ Concentration of AFM1(µg/L).

The initial AFM1concentration was 50 µg/L.

Mean ± standard deviation of triplicate samples.

Different superscript letters in the same column indicate a significant difference at p < 0.05.

D (day).

**Fig. 1:**
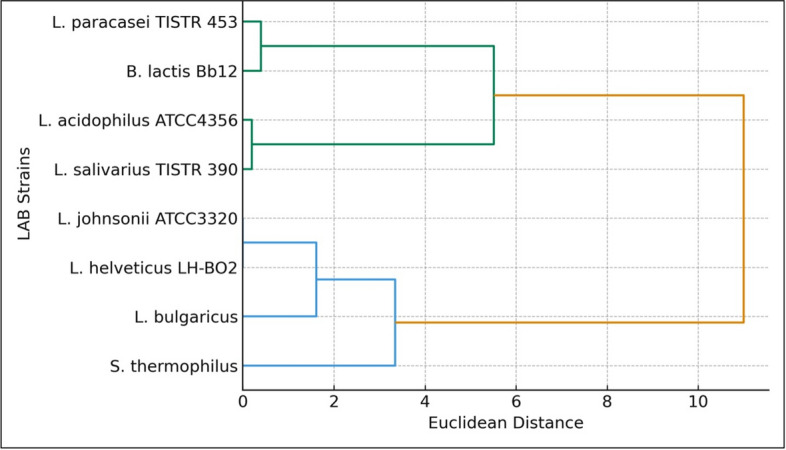
Hierarchical cluster analysis (HCA) of LAB strains based on their ability to remove AFM1 after 20 days of incubation in MRS broth. The dendrogram illustrates two major clusters: Cluster **I** includes *L. acidophilus* and *L. salivarius* as the most effective strains, while Cluster **II** comprises the remaining strains showing moderate to lower AFM1 removal efficiency.

Hierarchical cluster analysis (HCA) was performed to classify the eight LAB strains according to their efficiency in removing AFM1 after 20 days of incubation in MRS broth. The dendrogram revealed two major clusters. The first cluster grouped *L. acidophilus* ATCC4356 and *L. salivarius* TISTR 390, which exhibited the highest AFM1 removal rates of 80 and 79.8%, respectively, indicating their strong detoxification capacity. The second cluster included the remaining strains, with *S. thermophilus* (70.4%) and *L. bulgaricus* (72.2%) showing the lowest reduction levels (Fig. 1). These results indicate that *L. acidophilus* and *L. salivarius* are the most potent AFM1-reducing strains, showing a distinct clustering pattern that reflects their superior detoxification potential. This clustering pattern demonstrates clear variability among the tested LAB strains, suggesting that the ability to remove AFM1 is strain dependent. The close association between *L. acidophilus* and *L. salivarius* emphasizes their potential use as effective bio-detoxifying cultures for reducing AFM1contamination in dairy systems.

### Effect of mixed LAB starter cultures on AFM1 reduction and pH changes in Karish cheese during cold storage

Table 5 illustrates the effect of three combinations of LAB starter cultures on the removal of AFM1 from Karish cheese during processing and cold storage at 4 °C for 20 days. The data clearly show that all bacterial combinations were effective in progressively reducing AFM1 concentrations over time, accompanied by a consistent decrease in pH values, indicating enhanced acidification and metabolic activity of the cultures.

**Table 5:** Removal of AFM1 from Karishcheese using three different LAB strain combinations as starter cultures during processing and storage at 4°C.

*Starter culture*	pH/storage period										% AFM1 removal
	1 D		5 D		10 D		15 D		20 D		
	pH^#^	AFM1^@^	pH	AFM1	pH	AFM1	pH	AFM1	pH	AFM1	
*group No. 1*											
*S. thermophilus* EMCC-2											
*L. bulgaricus* EMCC-3	4.7	36.9 ± 1.6	4.3	28.8 ± 2.0	4.1	20.2 ± 0.4	3.9	17.5 ± 2.0	3.6	12.9 ± 1.5	74.2 ± 0.67^c^
*group No. 2*											
*S. thermophilus* EMCC-2											
*L. bulgaricus* EMCC-3											
*L. acidophilus* ATCC4356	4.6	34.8 ± 1.8	4.2	27.2 ± 1.0	4.0	20.1 ± 1.3	3.7	16.9 ± 0.3	3.4	11 ± 1.3	78.0 ± 1.03^a^
*group No. 3*											
*S. thermophilus* EMCC-2											
*L. bulgaricus* EMCC-3											
*L. salivarius* TISTR 390	4.7	33.4 ± 1.0	4.2	23.9 ± 1.8	4.0	19.4 ± 1.2	3.5	15.7 ± 1.7	3.3	11.8 ± 1.2	76.4 ± 1.39^b^

^**#**^measured pH value.

^**@**^Concentration of AFM1(µg/L).

The initial AFM1concentration was 50 µg/L.

Mean ± standard deviation of triplicate samples.

Different superscript letters in the same column indicate a significant difference at p < 0.05.

At the initial stage (1 day), the AFM1 content ranged from 36.9 µg/L in Group No. 1 (*S. thermophilus* + *L. bulgaricus*) to 33.4 µg/L in Group No. 3 (*S. thermophilus*, *L. bulgaricus,* and *L. salivarius*). As the incubation and storage progressed, the reduction in AFM1 became more pronounced. After 5 days, AFM1 concentrations declined to 28.8 µg/L, 27.2 µg/L, and 23.9 µg/L for Groups 1, 2, and 3, respectively, representing removal percentages of approximately 21.9%, 22.1%, and 28.5% compared to the initial 50 µg/L concentration. Over 10 days, the reduction trend continued, with AFM1 levels decreasing to 20.2 µg/L, 20.1 µg/L, and 19.4 µg/L for Groups 1, 2, and 3, respectively. The greatest detoxification efficiency was observed in Group No. 3, which achieved a 61.3% reduction at this stage. After 15 days, AFM1 concentrations dropped further to 17.5 µg/L, 16.9 µg/L, and 15.7 µg/L, while the pH values reached 3.9, 3.7, and 3.5, respectively. The lowest AFM1 value recorded at the final stage (20 days) was 11 µg/L for Group No. 2, followed closely by 11.9 µg/L for Group No. 3 and 12.9 µg/L for Group No. 1, corresponding to removal efficiencies of 78%, 76.4%, and 74.2%, respectively. Part of the initial reduction may be attributed to curd formation and whey drainage, whereas further decreases during storage are likely associated with LAB-mediated detoxification mechanisms.

## Discussion

Because of their outstanding safety record in food applications, lactic acid bacteria (LAB) are at the top of the list of microorganisms for mycotoxin degradation. LAB are favored over other microbes due to their many strains that are simple to cultivate and maintain, their natural growth in the human gut, which enables them to effectively eliminate mycotoxins, and their extreme safety for use in food^20^. Biodegradation ensures that toxins will be permanently detoxified, whereas adsorption allows for the potential release of these toxins in the gastrointestinal tract, thereby reducing the risks of systemic exposure. These considerations are especially important when considering fermented dairy products that have LAB as potential biocontrol agents for mycotoxins^17^. The LAB bacteria have two mechanisms for the detoxification of mycotoxins from foods. Panwar et al. also demonstrated that AFM₁ removal by *Lactobacillus* strains occurs predominantly through cell surface adsorption, with efficiency strongly influenced by strain specificity and viability status, supporting the adsorption-dominant mechanism observed in the present study^19^. Food detoxification by LAB is achieved using the viable cells of the microorganisms and/or using the enzymes produced by certain LAB strains^21^. Acids, carbon dioxide, hydrogen peroxide, phenyllactic acid, and bioactive low molecular weight peptides are examples of LAB bioactive molecules^22^. AFB1 and AFM1 have been researched for their structural features (the presence of a lactone ring and difuran moiety), which contribute to their mutagenic and carcinogenic properties. The progressive decrease in AFM₁ concentration observed in the present study is consistent with adsorption of the toxin onto LAB cell-surface components such as peptidoglycan, teichoic acids, and surface-layer proteins. Enzymatic degradation may additionally contribute in metabolically active cultures, but the present data, which lack LC–MS identification of degradation products, mass-balance verification, or desorption/recovery experiments, are insufficient to establish this. Accordingly, the mechanism is described here as adsorption-predominant, with enzymatic degradation noted as a possibility requiring future work^23^. The proposed enzymatic activities of LABs would include oxidoreductases, esterases, and peroxidases, which could catalyze reactions involving the opening of lactone rings, the reduction of double bonds located in the furan ring, and structural modifications to decrease toxin reactivity^24^. LAB generates a variety of proteolytic enzymes capable of hydrolyzing proteins, such as intracellular peptidases that break down the transferred peptides into amino acids, peptide transporters that carry the peptides into the cell, and cell-wall-bound proteinases that break down the protein into polypeptides^25^.

The structural changes can greatly reduce the biological activity of aflatoxins because they alter the sites where DNA can intercalate and sites for forming epoxide from aflatoxins. The degradation products of the aflatoxins from structural changes may be lower overall in terms of cytotoxicity and mutagenicity, but further toxicological evaluation of these degradation products is required to confirm this^26^. Biodegradation happens in varying degrees across different strains, but some of the most common strains proven to be effective in their ability to detoxify include, but are not limited to: *L. rhamnosus*, *L. plantarum*, and *L. casei.* These strains are also significantly influenced by environmental conditions such as pH level, incubation period, temperature, or initial concentration of toxins present. *Lactobacillus strains* tend to exhibit their highest levels of activity at a moderate acid pH range where both the bacteria will grow, and the enzymes will maintain a high degree of stability^27,28^. Biodegradation does not simply come from showing a reduction of toxic compounds as proof from a methodological perspective; therefore, more thorough proof must be provided by way of analytical testing to show that new metabolites were formed during the biodegradation process, using methods such as HPLC and LC–MS, to confirm that the chemical structure of the toxic compound had changed. Additionally, another line of evidence to support the theory that the toxic compound was actually degraded through the biodegradation process would be to show that aflatoxins were not released following multiple washings or gastrointestinal-like exposures^29^. In this study, eight different LAB strains were evaluated either alone or in combination to reduce AFM1 contamination in Karish cheese**.** Regarding oxygen requirements, the strain collection included both aerobic and anaerobic species, which is particularly relevant for assessing AFM1 detoxification mechanisms. Aerobic strains such as *L. helveticus*, *L. paracasei*, *L. acidophilus*, and *S. thermophilus* are typically associated with surface fermentation and rapid acidification, potentially enhancing AFM1 adsorption through cell wall charge modification. In contrast, anaerobic strains like *B. animalis subsp. lactis*, *L. salivarius*, *L. johnsonii*, and *L. bulgaricus* may rely more on metabolic or enzymatic degradation pathways that function optimally under low-oxygen environments. The inclusion of both homofermentative and heterofermentative LAB species allows for comparative analysis of AFM1 removal through distinct physiological routes—adsorption to cell wall components such as peptidoglycan and teichoic acids in viable or heat-killed cells, or possible enzymatic biotransformation in metabolically active strains^30^. Therefore, the composition of this bacterial panel provides a strong foundation for interpreting the subsequent results on AFM1 detoxification efficiency across different culture systems and storage conditions^30^.

Our findings also showed that viable cells are more effective in reducing AFM1 contamination as compared to the heat-killed cells. The reason for that is the heat likely alters cell wall structure (protein denaturation, polysaccharide rearrangement) and abolishes metabolic activity. Two non-exclusive effects are plausible, including: loss of active metabolic degradation pathways (so enzymatic breakdown is minimal); alteration of adsorption sites; and the heating may expose some hydrophobic sites in some strains (slightly improving passive adsorption), or conversely destroy/lower the affinity of ligand sites, reducing binding^31^.

The substantially lower removal compared with viable cells suggests that active physiological factors (cell surface properties maintained in viable cells, or metabolism-linked processes) play a major role in AFM1 removal observed with live cultures. These findings align with Panwar et al., who similarly reported a significant decline in AFM₁ binding capacity following heat inactivation of probiotic *Lactobacillus* strains^19^. According to the obtained results, the detoxification mechanism likely involves non-covalent adsorption of AFM1 molecules onto bacterial surface components such as peptidoglycans, polysaccharides, and teichoic acids. The greater efficiency of *L. acidophilus* could be attributed to its high surface area-to-volume ratio and hydrophobic cell surface, which enhance toxin binding^19^. Strains like *S. thermophilus* and *L. paracasei*, with smoother or less dense cell wall structures, showed lower binding potential. Moreover, since viable cells were used, partial metabolic degradation of AFM1 cannot be completely ruled out, particularly for species known to exhibit active enzymatic systems during incubation^19^.

All LAB strains tested can reduce AFM1 levels significantly, confirming their potential as biological adsorbents for mycotoxin decontamination. *L. acidophilus* ATCC 4356 is the most promising candidate, achieving over three-quarters removal under the tested conditions. The use of LAB in detoxification systems offers a safe, natural, and cost-effective alternative to chemical decontamination methods^1^. The trend of AFM1 removal in this study indicates that most of the toxin removal occurred within the first 8–16 h, followed by a slower rate of reduction up to 24 h. For example, *L. acidophilus* reduced AFM1 from 31.5 µg/L at 4 h to 18.5 µg/L at 16 h, and finally to 11.8 µg/L at 24 h, representing an incremental improvement of ~ 25% between 16 and 24 h. This plateau effect implies that extending incubation beyond 24 h may not yield significant additional detoxification.

A clear correlation was observed between pH decline and AFM1 reduction. For instance, in *L. acidophilus*, pH dropped from 4.5 (day 1) to 3.0 (day 20), while AFM1 fell from 31.5 µg/L to 10 µg/L. Similarly, *L. salivarius* showed a pH drop from 4.4 → 3.4 → 3.00, with AFM1 decreasing from 39.9 µg/L → 14.2 µg/L → 10.1 µg/L. This pattern indicates that the acidic environment produced by LAB metabolism enhances AFM1 binding or degradation. Lower pH values may promote electrostatic interactions between AFM1 molecules and bacterial cell wall components, facilitating adsorption. In the early stage (day 1–5): only partial removal is observed (typically 20–35%); metabolic activity is just beginning. Intermediate phase (day 10–15): marked acceleration in detoxification –AFM1reduced to ~ 35–40% of initial levels, coinciding with rapid acidification (pH ≈ 3.4–3.6). Late phase (day 20): near-maximum detoxification achieved (70–80% removal) as bacterial growth reached the stationary phase, and pH stabilized near 3.0. These results suggest that extended incubation enhances AFM1 removal due to cumulative adsorption and possible enzymatic modification over time.

LAB strains, especially *L. acidophilus*, *L. salivarius*, and *B. animalis subsp. lactis* demonstrates strong potential for biological detoxification of AFM1 in liquid matrices. The correlation between pH reduction and AFM1 removal supports the use of controlled fermentation as a safe, natural method to reduce mycotoxin contamination in dairy environments. However, long incubation times (≥ 15 days) may be impractical industrially; optimization of cell density, medium composition, and initial pH could accelerate detoxification. Regarding the effect of pH, the gradual decline in AFM1 concentration with the decrease in pH suggests a strong correlation between bacterial metabolic activity and toxin binding or degradation. The inclusion of *L. acidophilus* (Group No. 2) and *L. salivarius* (Group No. 3) appeared to enhance AFM1 removal compared with the traditional yogurt starter (*S. thermophilus* + *L. bulgaricus*). This improvement may be attributed to differences in cell wall composition and surface charge, which facilitate stronger adsorption of AFM1 molecules. Group No. 2 achieved the highest total reduction (78%) after 20 days, indicating the synergistic detoxifying activity of *L. acidophilus* when combined with the traditional starter bacteria^32,33^. It should be noted that the boiling treatment used to obtain non-viable cells may have altered certain cell-wall constituents, potentially exposing additional binding sites involved in AFM₁ adsorption. Therefore, differences observed between viable and heat-treated cells may reflect both the loss of metabolic activity and heat-induced modifications of surface structures.

## Conclusion

This study demonstrates that lactic acid bacteria (LAB) possess strong potential for the biological detoxification of aflatoxin M_1_ (AFM_1_) in dairy systems. Among the tested strains, Lactobacillus acidophilus ATCC 4356 showed the highest removal efficiency, followed by Bifidobacterium lactis Bb12 and Lactobacillus salivarius TISTR 390. Viable cells were significantly more effective than heat-killed cells, confirming the importance of cell viability and intact cell surface structures in AFM_1_ binding. AFM_1_ reduction was enhanced under acidic conditions and during prolonged fermentation, indicating a strong relationship between metabolic activity, pH decline, and detoxification efficiency. In Karish cheese, mixed starter cultures achieved up to ~ 78% AFM_1_ reduction during cold storage, demonstrating practical applicability in real dairy systems. Overall, selected LAB strains, particularly multi-strain starter combinations, offer a safe, natural, and effective strategy for reducing AFM_1_ contamination in fermented dairy products and enhancing food safety.

## Supplementary Information

Supplementary Information.

## Data Availability

All data generated or analyzed during this study are included in this published article and supplementary file.
