# Supplementary material for: Detoxification of aflatoxin M1 by eight lactic acid bacteria strains, alone or in combination, as viable and heat-killed cells in PBS, MRS broth, and Karish cheese
Source: Sci Rep. 2026 Aug 10;16:24729. doi: 10.1038/s41598-026-62660-7 (PMC13457558; doi:10.1038/s41598-026-62660-7)
Supplement: Supplementary file 1 [file 41598_2026_62660_MOESM1_ESM.pdf]

Journal: Applied Microbiology and Biotechnology

Type of Research: Original Research

Title; Detoxification of Aflatoxin M_1 by eight lactic acid bacteria as a single or as a mixed culture of viable and killed forms

Rafaat M. Elsanhoty[1], Mohammed Aladhadh[2], Mahmoud A. Al-Saman[1], Asmaa Abdella[1], Khaled M. Aboshanab[3*], Samar S. Mabrouk[4]

[1]Department of Industrial Biotechnology, Faculty of Biotechnology, University of Sadat City, Sadat City 22857/79, Egypt. rafaat.elsanhoty@gebri.usc.edu.eg; mahmoud.alsaman@gebri.usc.edu.eg; asmaa.abdelaal@gebri.usc.edu.eg

[2]Department of Food Science and Human Nutrition, College of Agriculture and Food, Qassim University, Buraydah 51452, Saudi Arabia. aladhadh@qu.edu.sa ; ORCID: 0000-0003-2070-5924

[3]Department of Microbiology and Immunology, Faculty of Pharmacy, Ain Shams University, Cairo 11566, Egypt

[4]Department of Microbiology, Faculty of Pharmacy, Ahram Canadian University (ACU), 6th October City, Giza 12566, Egypt. samar.mabrouk@acu.edu.eg

*Correspondence: Khaled M. Aboshanab - aboshanab2012@pharma.asu.edu.eg

https://orcid.org/0000-0002-7608-850X

Tel: (202)28434595

Mobile: +201007582620

Fax: (202)24051107

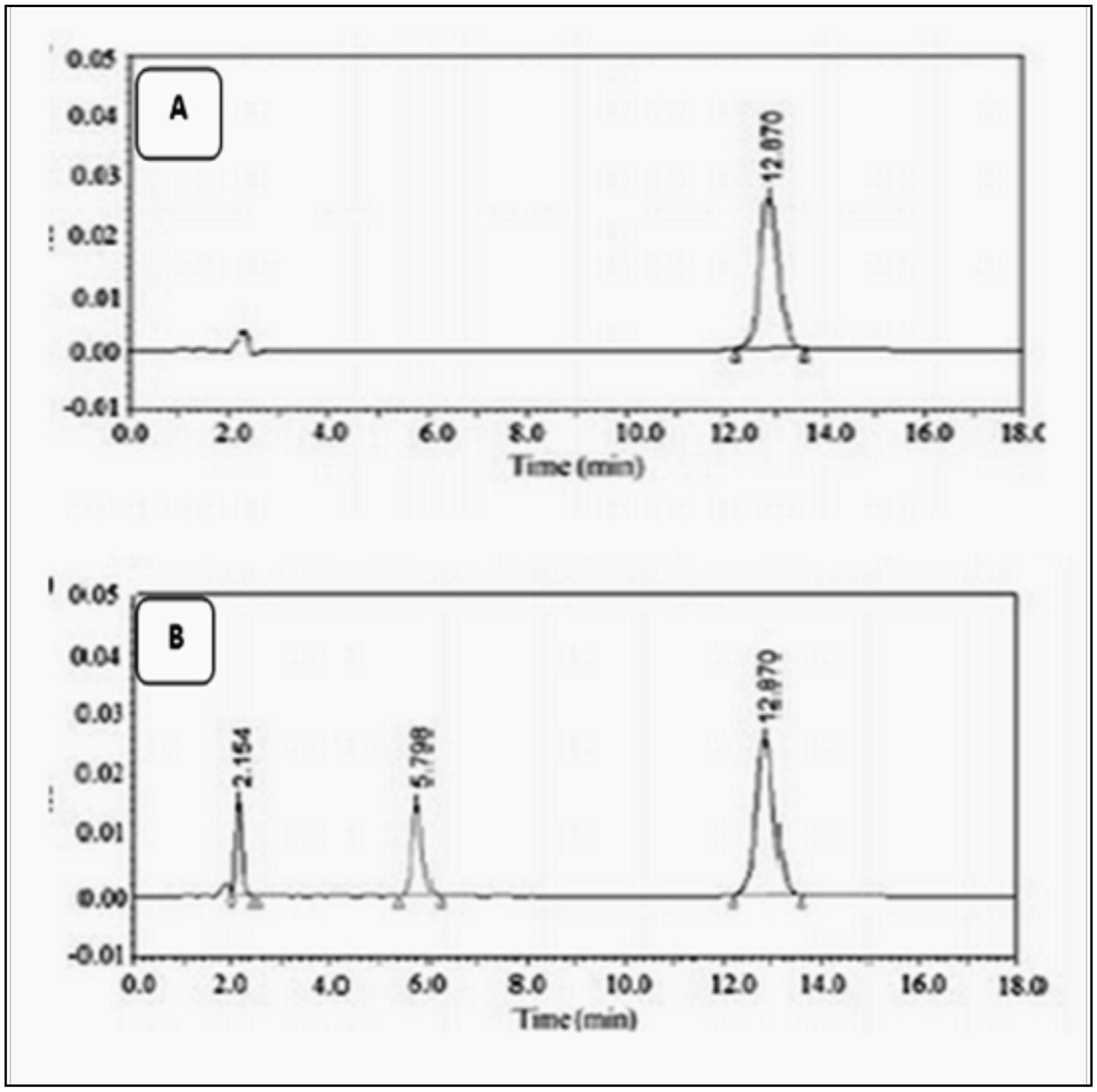

Fig. S1. HPLC chromatograms of A, AFM1 standard (3.6 ng mL^{-1}); B, sample of Karish cheese
